# The development of transversal competence of health service managers

**DOI:** 10.11606/s1518-8787.2019053001292

**Published:** 2019-09-02

**Authors:** Roberto Gonzalez Duarte, Irene Kazumi Miura, Namie Okino Sawada, Marilia Alves, Renata Petrin

**Affiliations:** I Universidade Federal de Minas Gerais. Faculdade de Ciências Econômicas. Departamento de Administração. Belo Horizonte, MG, Brasil; II Universidade de São Paulo. Faculdade de Economia, Administração e Contabilidade. Departamento de Administração. Ribeirão Preto, SP, Brasil; III Universidade Federal de Alfenas. Escola de Enfermagem. Alfenas, MG, Brasil; IV Universidade Federal de Minas Gerais. Escola de Enfermagem. Belo Horizonte, MG, Brasil; V Universidade Federal de Minas Gerais. Centro de Pós-Graduação e Pesquisas em Administração. Belo Horizonte, MG, Brasil

**Keywords:** Health Manager Competency-Based Education, Public Health, Higher Education, Coevolution

## Abstract

In their pedagogical projects, health management courses focus on multidisciplinarity, interdisciplinarity, comprehensiveness and transversality, whose main merit is to question health issues from different theoretical perspectives. Analyzing these issues under many perspectives does not necessarily mean the development of transversal competences. The development and application of these competences suppose going beyond integrating curricular contents and theory/practice. They depend on how the knowledge will be articulated to changes at organizational, sectorial and institutional levels and on the coevolution between these competences and changes. It is understood that health services manager role is effectively transversal when he/she: (i) acts at organizational boundaries, fostering interaction between organizations and other actors in the system; (ii) provides (and receives) feedback to (and from) these actors; and (iii) these feedbacks help decision makers to undertake organizational changes to respond to the environment and shape it.

## INTRODUCTION

The Restructuring and Expansion of Federal Universities program (REUNI) created 22 bachelors’ degrees in collective health and related denominations^a^ in higher education institutions (HEIs) all over Brazil. In their pedagogical projects, these courses focus on multidisciplinary training^b^ , interdisciplinarity^c^ , comprehensiveness of healthcare^c^ , transversality of knowledge in health^c^ , and articulation between theory and practice^b^ . In addition, they aim to train a general health manager, that is, with competences that allow them to act “in a reflexive, propositive and efficient manner in the many levels of management of the health system and services”^d^ (p.7) and “innovatively, humanistically and ethically committed to the health demands of the population”^b^ (p.21).

The main merit of multidisciplinarity, interdisciplinarity, comprehensiveness and transversality is to problematize health issues from different theoretical perspectives, from biological to social. This assumption, however, can be explained more by the transmission of knowledge from areas related to health (economy, administration, accounting, medicine, nursing, etc.) than by an effective integration of these types of knowledge. Analyzing health issues from several perspectives does not necessarily imply the development of transversal competences, defined as the integration between theoretical knowledge, skills and attitudes that contribute to an effective performance in different contexts^1^ . This development also depends on the coevolution between distinct knowledge and changes in the micro (organizational), meso (health sector) and macro (institutional) levels, that is, the mutual influence between them. It is discussed here how the coevolutionary perspective can contribute to understand the development of transversal competences.

### Multidisciplinarity and Transversality in the Health Area

The assumptions for education in the 21st century suggest that health professionals have a holistic approach, considering, in addition to biological processes, social determinants^2^ . In these professionals’ case, the construction of knowledge along with the health system is advocated in order to establish technical cooperation and qualify the management of SUS professionals^3^ .

Therefore, teaching in the health service area requires from teachers pedagogical skills to operationalize SUS principles, integrating practical and theoretical knowledge, indispensable to professional training. In this sense, the professional practice scenarios should be understood as spaces for the construction of new theories, and the production of knowledge in universities must be articulated with health units, responding to the social demands of these services^4^ . However, it should be observed that there are no formal spaces for this articulation yet. HEI is usually the reference of legitimate knowledge, not taking into account health services as learning spaces or producers of knowledge.

In opposition to this institutional model that values fragmented content, which, in turn, overvalues sequencing, transversality represents an alternative to these professionals’ formation. However, health education as a transversal proposal must break with the compartmentalization of knowledge, not simply inserting health issues in different activities of disciplines, treating them separately. Teachers and other professionals must joint efforts to develop actions to promote health in an integrated way.

Health service management courses are an adequate *locus* for the effective development of transversal competences. In addition to multidisciplinarity and interdisciplinarity, the radical macro and meso environmental transformations demand a different role of managers: acting at the organizational boundaries, articulating the organizational responses to the changes of these environments. These responses define the organization’s power to influence the environments where they are inserted. In the following sections, we examine these transformations in macro and meso environments and the need for a new look on the transversal competences of health service managers.

### Transformations and Challenges at the Health Area in Brazil

Since it is impossible to list all transformations at the macro and meso levels, some of the most significant ones are presented.

At the macro level, the change in the demographic and epidemiological profile^e^ is one of the most important transformations for the health sector. By 2025, the Brazilian population over 65 years old will be approximately 16% of the total population – the country will have the sixth largest older people population in the world. Even though public policies are defined to avoid the deleterious effects of this change on the health system, aging is naturally associated with an increase of chronic diseases (the prevalence of these diseases in the Brazilian population went from 29.9% in 2003 to 31.3% in 2008)^5^ . The radical nature of this change will have significant implications from the social point of view, since Brazil still has inadequate infrastructure to accommodate these patients. It will also financially affect individuals, families, organizations and public and private health systems. Researches show that health expenses grow faster than GDP, with relative increases of 50% to 100% depending on the country^6^ . The average care costs of a patient over 65 years old double in relation to those between 18 and 44 years old^7^ . Health financing in the future is unknown, and so are its solutions^8^ .

At the meso level, transformations, mainly technological–digitalization, *big data* , robotics, artificial intelligence, telemedicine, creation of startups^f^ , nanotechnology, DNA sequencing, among others–may be alternatives to solve some of the aforementioned problems. On the one hand, technological advances provoke interest because they are associated with longevity. On the other, these changes can mean increased costs for the health system. Some organizations are seeking to incorporate these new technologies in order to solve patient health and management issues. This incorporation, which illustrates how macro-environmental challenges can be faced considering technological change, depends on the transversal competences of health service managers.

### Coevolutionary Development of Transversal Competences

Transversality, one of the assumptions in the education of health service managers, is based on integrating curricular contents and theory/practice. It is necessary to go further, understanding it also from the manager’s role perspective, especially at the organizational boundaries’ level. The coevolution perspective helps to understand how this activity in the interaction spaces between micro, meso and macro levels is essential for the development of transversal competence. This perspective analyzes how organizations, sectors and institutions develop over time and how they influence each other^9^ . Coevolutionary studies also discuss the managerial intentionality role, on the one hand, for the adaptation of organizations^10^ and, on the other, for the modification of their environments^11^ .

For example, when analyzing the role of individuals in coevolution between academic disciplines and an industry segment, Murmann (2013) observes how the interchange between academic institutions and organizations (two-way causal mechanism) had consequences for both. According to this author, “from a coevolutionary perspective, human agents can to some extent shape their own environment”^12^ (p.75). From this perspective, when one expands the idea of transversality beyond the integration between disciplinary content and theory and practice, and considering the assumptions of micro-meso-macro interaction, of mutual influence and of managerial intentionality, it is understood that the role of the health services’ manager is effectively transversal when he/she: (i) acts in the organizational boundaries, fostering interaction between the organizations and other actors in the system; (ii) provides (and receives) feedback to (and from) organizations, government institutions, industry associations, universities, among others; and (iii) these feedbacks help decision makers to undertake organizational change to respond to the environment and, at the same time, shape it.

There are, however, some challenges to this action. At the macro level, public policies for health are conditioned by political-governmental priorities and by the financial availability of individuals, families and public power. At the meso level, the main challenges are to foster the development of technologies and innovations directed to the health area and, above all, if and how) they can be used to improve the management of the public health system, ensuring its sustainability. At the micro level, the challenge is the effective incorporation of these innovations and technologies by organizations. Health service managers will be, thus, key players for the functioning of the governance among these three levels.

## FINAL CONSIDERATIONS

Despite the pioneering initiative of some public universities to create health service management courses, some of their assumptions need to be adjusted due to the transformations in macro and meso environments. Considering the assumptions from the coevolutionary perspective, and particularly the fact that the universities and the public and private organizations of the system influence each other, the development of transversal competences, a fundamental idea of these bachelor’s degrees, must be reviewed. This development is not limited to the integration of academic contents or between theory and practice, but supposes that the actions of these managers are influenced by transformations in the macro and meso environments and can influence organizational adaptation and survival, thus affecting other organizations and the system itself.

Therefore, the university can have a fundamental role in building transversal competences more adequate to the environmental transformations of Brazilian public health. Considering the institutional constraints of public universities, this construction supposes first to redefine the roles (governance) and places (organizational boundaries) of health managers; then to increase the interaction with the other actors of the system, receiving, and, at the same time, providing feedback that will subsidize the construction of this competence; and, finally, to assess how the meso and macro environmental transformations can influence the formation of these managers, especially their transversal competences.

## Footnotes

a Associação Brasileira de Saúde Coletiva. Cursos. Rio de Janeiro: ABRASCO; c2018 [cited 2018 Sep 1]. Available from: https://www.abrasco.org.br/site/graduacaoemsaude/cursos

b Universidade Federal de Minas Gerais. Bacharelado em Gestão de Serviços de Saúde: projeto pedagógico. Belo Horizonte; 2018 [cited 2018 Sep 8]. Available from: http://www.enf.ufmg.br/index.php/projeto-pedagogico/365-projeto-pedagogico-1/file

c Universidade de Brasília, Faculdade de Ceilândia. Curso de Graduação em Saúde Coletiva: projeto político-pedagógico. Brasília, DF; 2009 [cited 2018 Sep 8]. Available from: http://fce.unb.br/images/documentos/graduacao/saudecoletiva/ppp/ppp_sc_19-08.pdf

d Universidade Federal do Rio Grande do Norte, Centro de Ciências da Saúde. Curso de Saúde Coletiva: projeto pedagógico do curso. Natal; c2006/2019 [cited 2018 Sep 8]. Available from: https://sigaa.ufrn.br/sigaa/public/curso/ppp.jsf?lc=pt_BR&id=7252420

e Alisson E. Brasil terá sexta maior população de idosos do mundo até 2025. Agência FAPESP. 2016 Jul 6 [cited 2018 Oct 20]. Available from: http://agencia.fapesp.br/brasil-tera-sexta-maior-populacao-de-idosos-no-mundo-ate-2025/23513/

f Associação Brasileira de Startups. O momento da startup brasileira e o futuro do ecossistema de inovação. São Paulo: abstartups; 2017 [cited 2018 Oct 20]. Available from: https://abstartups.com.br/PDF/radiog.rafia-startups-brasileiras.pdf
