# Supplementary material for: The development of transversal competence of health service managers
Source: Rev Saude Publica. 2019 Sep 2;53:74. doi: 10.11606/s1518-8787.2019053001292 (PMC6758847; doi:10.11606/s1518-8787.2019053001292)
Supplement: Translation

# Translation: O desenvolvimento da competência transversal dos gestores de serviços de saúde

**Authors:** Roberto Gonzalez Duarte, Irene Kazumi Miura, Namie Okino Sawada, Marilia Alves, Renata Petrin

**Affiliations:** I Universidade Federal de Minas Gerais. Faculdade de Ciências Econômicas. Departamento de Administração. Belo Horizonte, MG, Brasil; II Universidade de São Paulo. Faculdade de Economia, Administração e Contabilidade. Departamento de Administração. Ribeirão Preto, SP, Brasil; III Universidade Federal de Alfenas. Escola de Enfermagem. Alfenas, MG, Brasil; IV Universidade Federal de Minas Gerais. Escola de Enfermagem. Belo Horizonte, MG, Brasil; V Universidade Federal de Minas Gerais. Centro de Pós-Graduação e Pesquisas em Administração. Belo Horizonte, MG, Brasil

**Keywords:** Gestor de Saúde, Educação Baseada em Competências, Saúde Pública, Educação Superior, Coevolução

## Abstract

Em seus projetos pedagógicos, os cursos de gestão de saúde privilegiam a multidisciplinaridade, interdisciplinaridade, integralidade e transversalidade, cujo principal mérito é problematizar as questões da saúde sob diferentes perspectivas teóricas. Analisar as questões da área da saúde a partir de diversos prismas não implica necessariamente no desenvolvimento de competências transversais. O desenvolvimento e a aplicação dessas competências pressupõem ir além da integração entre conteúdos curriculares e entre teoria e prática. Dependem de como os conhecimentos serão articulados às mudanças nos níveis organizacional, setorial e institucional e da coevolução entre essas competências e essas mudanças. Entende-se que a atuação do gestor de serviços de saúde é efetivamente transversal quando: (i) atua nas fronteiras organizacionais, fomentando a interação entre as organizações e outros atores do sistema; (ii) provê (e recebe) *feedbacks* para esses (desses) atores; e (iii) esses *feedbacks* auxiliam os tomadores de decisão a empreender mudanças organizacionais, de modo a responder ao ambiente e a moldá-lo.

## INTRODUÇÃO

O programa Reestruturação e Expansão das Universidades Federais (REUNI) deu lugar à criação de 22 bacharelados em saúde coletiva e denominações afins^a^ em instituições superiores de ensino (IES) em todas as regiões do Brasil. Em seus projetos pedagógicos, esses cursos privilegiam a formação multidisciplinar^b^ , interdisciplinaridade^c^ , integralidade da atenção à saúde^c^ , transversalidade do saber em saúde^c^ e articulação entre teoria e prática^b^ . Além disso, têm como objetivo formar um gestor da saúde generalista, i.e., com competências que o permitam atuar “de forma reflexiva, propositiva e eficiente nos diversos níveis de gestão do sistema e dos serviços de saúde”^d^ (p.7) e de “maneira inovadora, humanista e eticamente comprometida com as demandas de saúde da população”^b^ (p.21).

O principal mérito da multidisciplinaridade, interdisciplinaridade, integralidade e transversalidade é problematizar as questões da saúde a partir de perspectivas teóricas distintas, do biológico ao social. Esse pressuposto se traduz, entretanto, mais pela transmissão de conhecimentos de áreas correlatas à saúde (economia, administração, contabilidade, medicina, enfermagem etc.) do que pela efetiva integração entre esses saberes. Analisar as questões da área da saúde a partir de diversos prismas não implica necessariamente no desenvolvimento de competências transversais, definidas como a integração entre conhecimentos teóricos, habilidades e atitudes que contribuem para uma atuação eficaz em diferentes contextos^1^ . Esse desenvolvimento depende também da coevolução entre os distintos conhecimentos e as mudanças nos níveis micro (organizacional), meso (setor de saúde) e macro (institucional), i.e., da influência mútua entre ambos. Discute-se, aqui, como a perspectiva coevolutiva pode contribuir para se compreender o desenvolvimento das competências transversais.

### Multidisciplinaridade e Transversalidade na Área da Saúde

As diretrizes para a educação no século XXI sugerem que os profissionais de saúde tenham uma visão holística, considerando, além dos processos biológicos, os condicionantes sociais^2^ . No caso desses profissionais, preconiza-se a construção do ensino junto com o sistema de saúde, a fim de se estabelecer a cooperação técnica para qualificar a gestão dos profissionais na rede SUS^3^ .

O ensino na área de serviço de saúde requer dos docentes, portanto, competências pedagógicas para operacionalizar os princípios do SUS, integrando os saberes da prática e da teoria, indispensáveis à formação profissional. Nesse sentido, os cenários da prática devem ser entendidos como um espaço de construção de novas teorias, e a produção do conhecimento nas universidades deve ser articulada com as unidades de saúde, respondendo às demandas sociais desses serviços^4^ . Observe-se, contudo, que ainda não existem espaços formais para essa articulação. Geralmente, a IES é referência do saber legítimo, não levando em conta os serviços de saúde como espaços de aprendizagem ou produtores de conhecimento.

Em contraposição ao modelo institucional que valoriza conteúdos fragmentados, que por sua vez supervalorizam a sequenciação, a transversalidade representa uma alternativa para a formação desses profissionais. Contudo, a educação em saúde como proposta transversal deve romper com a compartimentalização do conhecimento, não bastando simplesmente a inserção de temas de saúde em diversas atividades das disciplinas escolares, em que cada professor os trata de forma isolada. É necessário um esforço conjunto, no qual professores e outros profissionais irão desenvolver ações buscando promover a saúde de forma integrada.

Os cursos de gestão de serviços de saúde constituem um *locus* adequado para o efetivo desenvolvimento de competências transversais. Para além da multidisciplinaridade e interdisciplinaridade, as radicais transformações macro e meso ambientais demandam uma atuação do gestor de outra ordem, nas fronteiras das organizações, articulando as respostas organizacionais às transformações desses ambientes. Essas respostas definem o poder da organização de influenciar os ambientes em que estão inseridas. Nas seções seguintes, examinam-se essas transformações nos ambientes macro e meso e a necessidade de um novo olhar sobre as competências transversais dos gestores de serviços de saúde.

### Transformações e Desafios na Área da Saúde no Brasil

Não sendo possível listar neste espaço todas as transformações nos níveis macro e meso, apresentam-se algumas entre as mais significativas.

No nível macro, uma das mais importantes para o setor de saúde é a mudança do perfil demográfico e epidemiológico^e^ . Em 2025, a população brasileira acima de 65 anos corresponderá a aproximadamente 16% da população total – o país terá a sexta maior população idosa do mundo^f^ . Mesmo que políticas públicas sejam definidas para se evitar os efeitos deletérios dessa mudança para o sistema de saúde, o envelhecimento está naturalmente associado com o aumento de doenças crônicas (a prevalência dessas doenças na população brasileira cresceu de 29,9%, em 2003, para 31,3%, em 2008)^5^ . A radicalidade dessa mudança terá implicações não negligenciáveis do ponto de vista social, uma vez que o Brasil ainda não tem uma infraestrutura adequada para acolher esses pacientes, bem como do ponto de vista financeiro para os indivíduos, as famílias, as organizações e os sistemas de saúde público e privado. Pesquisas mostram que os gastos com saúde crescem mais rápido que o PIB, com aumentos relativos de 50% a 100%, dependendo do país^6^ . Os custos assistenciais médios de um paciente acima de 65 anos dobram em relação àqueles entre 18 e 44 anos^7^ . O financiamento da saúde no futuro representa uma incógnita cujas soluções ainda não parecem claras^8^ .

No nível meso, transformações, principalmente tecnológicas – digitalização, *big data* , robótica, inteligência artificial, telemedicina, criação de *startups* ^f^ , nanotecnologia, sequenciamento de DNA, entre outras – podem ser alternativas para se solucionar alguns dos problemas apontados acima. Por um lado, avanços tecnológicos provocam interesse, porque estão associados à vida mais longeva. Por outro lado, essas mudanças podem significar aumento de custos para o sistema de saúde. Algumas organizações buscam incorporar essas novas tecnologias de maneira a solucionar as questões relativas à saúde dos pacientes e também à gestão. Essa incorporação, que ilustra como os desafios macro ambientais poderão ser enfrentados a partir das transformações tecnológicas, depende em certa medida das competências transversais dos gestores de serviços de saúde.

### Desenvolvimento Coevolutivo de Competências Transversais

A transversalidade, um dos pressupostos na formação dos gestores de serviços de saúde, baseia-se na integração entre conteúdos curriculares e entre teoria e prática. Argumenta-se que é necessário ir além, entendendo-a também a partir da atuação do gestor, sobretudo nas fronteiras da organização. A perspectiva da coevolução auxilia a compreender como essa atuação nos espaços de interação entre os níveis micro, meso e macro é essencial para o desenvolvimento da competência transversal. Essa perspectiva analisa como organizações, setores e instituições se desenvolvem ao longo do tempo e se influenciam mutuamente^9^ . Os estudos coevolutivos discutem também o papel da intencionalidade gerencial, por um lado, para a adaptação das organizações^10^ e, por outro, para a modificação de seus ambientes^11^ .

Murmann (2013), por exemplo, ao analisar o papel dos indivíduos na coevolução entre disciplinas acadêmicas e um segmento da indústria, observa como o intercâmbio entre as instituições acadêmicas e as organizações (mecanismo causal bidirecional) teve consequências para ambas. Segundo esse autor, “do ponto de vista da perspectiva coevolutiva, os agentes humanos podem moldar seus próprios ambientes”^12^ (p.75). Tendo essa perspectiva como referência, ao se ampliar a ideia de transversalidade para além da integração entre conteúdos disciplinares e entre teoria e prática, e levando em conta os pressupostos da interação micro-meso-macro da influência mútua e da intencionalidade gerencial, entende-se que a atuação do gestor de serviços de saúde é efetivamente transversal quando: (i) atua nas fronteiras organizacionais, fomentando a interação entre as organizações e outros atores do sistema; (ii) provê (e recebe) *feedbacks* para (das) organizações, instituições governamentais, associações setoriais, universidades, entre outros; e (iii) esses *feedbacks* auxiliam os tomadores de decisão a empreender mudanças organizacionais, de modo a responder ao ambiente e, ao mesmo tempo, moldá-lo.

Há, entretanto, alguns desafios a essa atuação. No nível macro, as políticas públicas para a área da saúde são condicionadas pelas prioridades político-governamentais e pelas disponibilidades financeiras dos indivíduos, das famílias e do poder público. No nível meso, os principais desafios são fomentar o desenvolvimento de tecnologias e inovações dirigidas à área da saúde e, sobretudo, se (e como) elas podem ser utilizadas para o aperfeiçoamento da gestão do sistema público de saúde, assegurando a sua sustentabilidade. No nível micro, o desafio é a efetiva incorporação dessas inovações e tecnologias pelas organizações. Os gestores de serviços de saúde serão, assim, atores-chave para o funcionamento dessa governança entre os três níveis.

## CONSIDERAÇÕES FINAIS

Apesar da iniciativa pioneira de algumas universidades públicas na criação de cursos de gestão de serviço de saúde, alguns de seus pressupostos precisam ser ajustados em função das transformações nos ambientes macro e meso. Levando-se em conta os pressupostos da perspectiva da coevolução e, particularmente, o fato de que as universidades e as organizações (públicas e privadas) do sistema se influenciam mutuamente, o desenvolvimento da competência transversal, noção fundamental dessas graduações, deve ser revista. Esse desenvolvimento não se reduz à integração de conteúdos acadêmicos ou entre teoria e prática, mas pressupõe que as ações desses gestores são influenciadas pelas transformações nos ambientes macro e meso e podem influenciar a adaptação e sobrevivência organizacionais, afetando assim outras organizações e o próprio sistema.

A universidade pode, portanto, ter um papel fundamental na construção de competências transversais mais adequadas às transformações ambientais da saúde pública brasileira. Considerando-se os limites institucionais das universidades públicas, essa construção pressupõe primeiro redefinir os papéis (governança) e os lugares (fronteiras organizacionais) de atuação dos gestores de saúde; depois, incrementar a interação com os outros atores do sistema, recebendo e ao mesmo tempo provendo *feedbacks* que subsidiarão a construção dessa competência; e por último, avaliar como as transformações meso e macro ambientais podem influenciar a formação desses gestores, sobretudo suas competências transversais.

## Footnotes

a Associação Brasileira de Saúde Coletiva. Cursos. Rio de Janeiro: ABRASCO; c2018 [citado 1 out 2018]. Disponível em: https://www.abrasco.org.br/site/graduacaoemsaude/cursos

b Universidade Federal de Minas Gerais. Bacharelado em Gestão de Serviços de Saúde: projeto pedagógico. Belo Horizonte; 2018 [citado 8 set 2018]. Disponível em: http://www.enf.ufmg.br/index.php/projeto-pedagogico/365-projeto-pedagogico-1/file

c Universidade de Brasília, Faculdade de Ceilândia. Curso de Graduação em Saúde Coletiva: projeto político-pedagógico. Brasília, DF; 2009 [citado 8 set 2018]. Disponível em: http://fce.unb.br/images/documentos/graduacao/saudecoletiva/ppp/ppp_sc_19-08.pdf

d Universidade Federal do Rio Grande do Norte, Centro de Ciências da Saúde. Curso de Saúde Coletiva: projeto pedagógico do curso. Natal; c2006/2019 [citado 8 set 2018]. Disponível em: https://sigaa.ufrn.br/sigaa/public/curso/ppp.jsf?lc=pt_BR&id=7252420

e Alisson E. Brasil terá sexta maior população de idosos do mundo até 2025. Agência FAPESP. 6 jul 2016 [citado 20 out 2018]. Disponível em: http://agencia.fapesp.br/brasil-tera-sexta-maior-populacao-de-idosos-no-mundo-ate-2025/23513/

f Associação Brasileira de Startups. O momento da startup brasileira e o futuro do ecossistema de inovação. São Paulo: abstartups; 2017 [citado 20 out 2018]. Disponível em: https://abstartups.com.br/PDF/radiog.rafia-startups-brasileiras.pdf
